# Network Entropy for the Sequence Analysis of Functional Connectivity Graphs of the Brain

**DOI:** 10.3390/e20050311

**Published:** 2018-04-25

**Authors:** Chi Zhang, Fengyu Cong, Tuomo Kujala, Wenya Liu, Jia Liu, Tiina Parviainen, Tapani Ristaniemi

**Affiliations:** 1 School of Biomedical Engineering, Faculty of Electronic Information and Electrical Engineering, Dalian University of Technology, Dalian 116024, China; 2 Faculty of Information Technology, University of Jyvaskyla, Jyvaskyla FIN-40014, Finland; 3 Department of Psychology, University of Jyvaskyla, Jyvaskyla FIN-40014, Finland

**Keywords:** network entropy, connectivity, brain network, dynamic network analysis, event-related analysis, driver fatigue

## Abstract

Dynamic representation of functional brain networks involved in the sequence analysis of functional connectivity graphs of the brain (FCGB) gains advances in uncovering evolved interaction mechanisms. However, most of the networks, even the event-related ones, are highly heterogeneous due to spurious interactions, which bring challenges to revealing the change patterns of interactive information in the complex dynamic process. In this paper, we propose a network entropy (NE) method to measure connectivity uncertainty of FCGB sequences to alleviate the spurious interaction problem in dynamic network analysis to realize associations with different events during a complex cognitive task. The proposed dynamic analysis approach calculated the adjacency matrices from ongoing electroencephalpgram (EEG) in a sliding time-window to form the FCGB sequences. The probability distribution of Shannon entropy was replaced by the connection sequence distribution to measure the uncertainty of FCGB constituting NE. Without averaging, we used time frequency transform of the NE of FCGB sequences to analyze the event-related changes in oscillatory activity in the single-trial traces during the complex cognitive process of driving. Finally, the results of a verification experiment showed that the NE of the FCGB sequences has a certain time-locked performance for different events related to driver fatigue in a prolonged driving task. The time errors between the extracted time of high-power NE and the recorded time of event occurrence were distributed within the range [−30 s, 30 s] and 90.1% of the time errors were distributed within the range [−10 s, 10 s]. The high correlation (*r* = 0.99997, *p* < 0.001) between the timing characteristics of the two types of signals indicates that the NE can reflect the actual dynamic interaction states of brain. Thus, the method may have potential implications for cognitive studies and for the detection of physiological states.

## 1. Introduction

Imaging of functional connectivity provides an effective means to describe the dependencies of the functional activities among different brain regions [1]. With the introduction of graph theory, the functional connectivity in brain networks can be imaged as a functional connectivity graph of the brain (FCGB), which has become increasingly useful for revealing the underlying cognitive mechanisms and abnormally interactive activities in several psychiatric and neurological disorders [2,3]. Since the topological structure of FCGB has a great influence on its convergence performance, assessment and quantification of the topological structure is a fundamental issue in connectivity studies [1,4].

One of the conventional and commonly-used approaches to attacking this problem is extracting the characteristics of a small-world network [5,6,7,8]. The small-world features are determined by two factors: the local interconnectedness and overall connectedness of the graph reflected by cluster coefficient and characteristic path length (i.e., average path length) respectively. Densely interconnected neighbors (nodes/brain regions) yield high clustering coefficients and have larger resilience [9]. If the characteristic path length is smaller, then the potential for integration will be stronger, because of the fewer links that need to be traversed to go from one arbitrary node to another. The small-world networks are known as graphs with many local connections and a few random long distance connections, so they are near-optimal networks and the small-world features can be used to assess the intrinsic properties of FCGB considering the efficiency and cost simultaneously [10]. For example, according to Delbeuck et al. [11], cognitive dysfunction in Alzheimer’s disease (AD) could be due, at least in part, to a functional disconnection between distant brain areas. Further, Stam et al. [10] showed a longer path length with relatively preserved cluster coefficients in Alzheimer’s patients, which suggests a loss of efficiency with less optimal organization and provides support for the presence of small-world features in FCGBs. Vecchio et al. [12] also found a high statistical correlation between small-world features and memory performance. Namely, an increase of small worldness in a specific frequency band during the resting state is linked to a better performance in short-term memory as evaluated by digit span tests. Sun et al. [13] revealed increasing changes of characteristic path length in a mentally-demanding test of sustained attention, providing further support for the presence of a reshaped global topology in FCGBs under fatigue state. 

In addition, as evaluation ability based on just cluster coefficient and characteristic path length is limited, some studies introduced more indicators from graph theory to provide richer information about the topological properties. For instance, Fraschini et al. [14] adopted a weighted clustering coefficient, a weighted characteristic path length and minimum spanning tree (MST) parameters to evaluate the FCGB topology under the condition of different epochs with variable length. The results showed that the source-level MST parameters were less sensitive to differences in epoch length, which is beneficial for comparing topological properties between different studies. As well as small-worldness, global efficiency, local efficiency and vulnerability were also utilized to analyze the statistical features of topology structure [15]. The efficiency indicators reflect how efficiently the nodes in the graphs exchange information [16]. Vulnerability was proposed as a method for measuring damage of connectivity when removing nodes of the graph [17]. Therefore, the assessment and quantification methods of the topological structure are becoming more and more comprehensive. However, most of them are static methods and are based on statistical analysis at different stages or in different conditions [13,14,18,19]. It is still hard to achieve dynamic representation and event-related assessment in single-trial traces throughout the whole complex process. Sometimes the dynamic information may not be reliable in practice [20], especially for electroencephalography (EEG)-based connectivity analysis. 

EEG is a measure to record brain electrical activities non-invasively at a millisecond time scale and in some circumstances single-trial EEG analysis has proven able to reveal more temporal component features than averaged event related potential (ERP) [21,22], thus making it a potential tool for investigating the neural dynamics of complex cognitive processes within spatially well-defined neural networks [23]. Because connectivity calculated based on ongoing EEG has a relatively low signal-to-noise ratio (SNR) in single-trial traces, it is difficult to extract the event-related dynamic information from different FCGBs with a reliable link to cognitive processes [20]. Additionally, FCGBs estimated from EEG sensor space data are neuroanatomically uninformative and severely confounded by signal mixing (a mixture of signals from several distinct sources detected by one sensor) [24]. Functional connectivity measures usually suffer from two types of false positives—artificial and spurious interactions [25]. Artificial interactions arise directly from signal mixing, in which one true signal is smeared to multiple sensors or sources, regardless of whether true interactions are present. It can be suppressed by a number of binarized connectivity matrices that typically aim to remove linear coupling terms and therefore remove some artificial interactions [26]. Spurious interactions—referred to as ghost interactions—arise from the leakage of signals from a true link of sources to the surrounding links. The issue of spurious interactions is more difficult to solve because of multivariate mixing effects [24]. Up to now, steps towards addressing this problem have been taken for correcting spurious interactions in oscillation amplitude correlation estimates [27,28]. Nevertheless, the connectivity matrices are estimated at source-level and no solution has been proposed to suppress spurious interactions in a dynamic network analysis.

To further address this problem, we introduce estimators from information theory for extending the manifestations of graph theory. In information theory, entropy measurements play a key role in representing complexity and uncertainty through the probability distributions that underlie the process of communication [29,30]. Various studies have proven that the entropy-based algorithms are useful and robust estimators for evaluating regularity or predictability according to bioelectric/biomagnetic signals [31,32,33,34,35].

In this paper, we present a network entropy (NE) method for quantifying and assessing the topological properties of FCGB sequences to realize event-related dynamic analysis throughout a complex process. Instead of excluding the contribution of instantaneous signal spread to the estimated interactions (leakage insensitive measurements), Shannon entropy (SE) was calculated to measure the connectivity uncertainty of FCGBs in every epoch. Taking multivariate mixing effects into account, the nonlinear dynamical features were resolved in the time-frequency domain by using wavelet time-frequency transform so that we can analyze the induced responses or detect the physiological states with robust performance.

## 2. Materials and Methods 

### 2.1. Sequence Analysis of FCGBs

**Figure 1:**
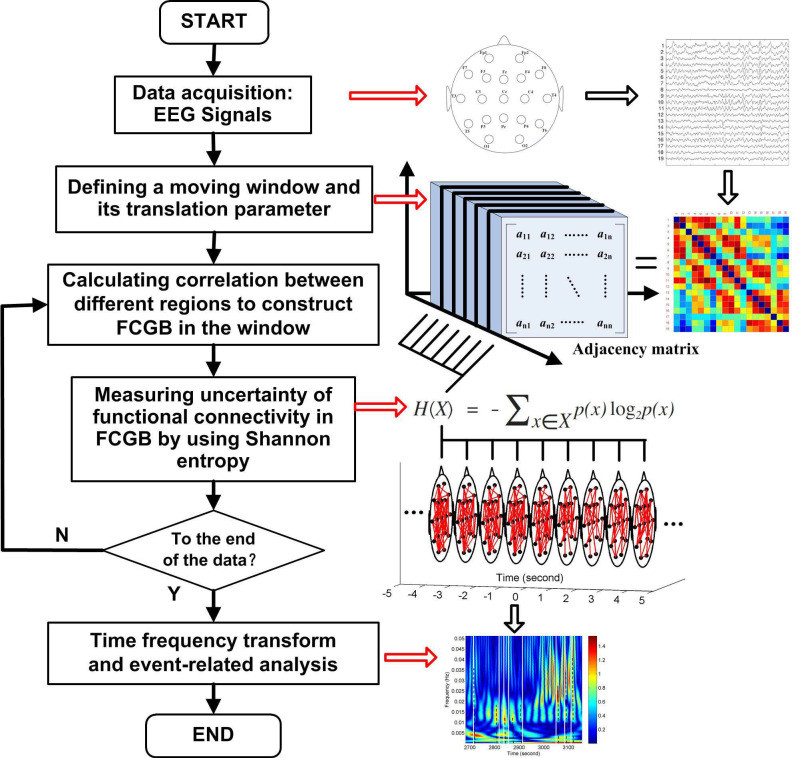
Flowchart for the research approach presented in the paper.

Figure 1 shows a flowchart for the research approach presented in this paper. The NE method starts at the step after sliding window settings which are used to get FCGB sequences. The window is moved step by step within the data range through a shift factor *m*. Every time the window is moved a step, functional connectivity is estimated to construct a FCGB and the SE algorithm is implemented to quantify the uncertainty in the global topographical network. After the window is moved to the last part of the data, the loop in Figure 1 is broken and time frequency transform is conducted to realize the event-related analysis throughout the whole process. 

According to graph theory, constructing FCGB requires the identification of nodes and edges [36]. Because of the advantages of time resolution and practicality, in this paper, we established FCGB based on EEG signals. EEG electrodes were assigned to the nodes of FCGB. The adjacency relations among the nodes in FCGB can be described by the functional connectivity matrix ***F***, whose element ***F***(*i*, *j*), shows the measured edge between electrodes (nodes) *i* and *j*. There are large numbers of edge measurement methods divided into time domain methods such as cross-correlation function [37] and synchronization likelihood [38] and frequency or phase domain methods such as coherence, phase synchronization [39] and phase lag index [40]. Even though previous studies have shown that the correlation-based and coherence-based methods suffer from the primary and secondary leakage which may lead to false positives due to “ghost interactions” [26], the solution has already been proposed in the case of amplitude correlations [27]. Sliding-window correlation estimates have also been used to assess the uncertainty underlying dynamic connectivity by providing confidence bands [41]. Therefore, a sliding-window cross-correlation function was adopted in this study to reflect the dynamic adjacency relations among different node pairs.

In the sliding window (each epoch), the correlation between EEG signals *s_i_* and *s_j_* can be calculated by the following formulas:

(1) $$\gamma_{ij}=\left|\frac{CC(s_{i},s_{j})(\tau )}{\sqrt{CC(s_{i},s_{i})(0)CC(s_{j},s_{j})(0)}}\right|,$$

(2) $$CC(s_{i},s_{j})(\tau )=\{\begin{array}{ll} \sum_{t=1}^{N-\tau }s_{i}(t+\tau )s_{j}(t), & \tau \geq 0\\ CC(s_{j},s_{i})(-\tau ), & \tau <0 \end{array},$$

where the equal-time cross-correlation function was chosen, that is, *τ* = 0. *γ_ij_* corresponds to the element of the functional connectivity matrix ***F***, which presents in *i-*th row and *j-*th column. To exclude self-connections of nodes, the elements on the main diagonal of ***F*** were set to zero. The off-diagonal value of ***F*** varies between 0, meaning no correlation and 1 meaning complete correlation and interdependency between pairs of EEG electrodes. Here, we employed the sliding window with the translation parameter to be sampling length (SL) to calculate the time-resolved cross-correlation matrices and form FCGB sequences at the second time scale. The window moved along the data points step by step and the functional connectivity matrices constituted a tensor **F**, whose element **F**(***F***, *k*) shows the constructed FCGB in epoch *k*. The following step is in connection with the assessment and quantification of the **F** properties. 

Since a drawback of the sliding-window correlation technique is that spurious fluctuations caused by noise can easily be confused with task-related signal, the ability to assess the level of uncertainty in sliding-window correlation estimates is of critical importance [41]. To quantify the uncertainty of interactive information in FCGBs, we consider an FCGB as a discrete information source *X* that has a set of possible interactive activities whose probabilities of occurrence are *p*_l_, *p*_2_, ..., *p_n_*. Thus, we have

(3) $$\left[\begin{array}{c} X\\ p(x) \end{array}\right]=\left[\begin{array}{ccccc} x_{1} & x_{2} & \cdots & x_{n-1} & x_{n}\\ p_{1} & p_{2} & \cdots & p_{n-1} & p_{n} \end{array}\right],\;\sum_{i=1}^{n}p_{i}=1,$$

where *x*_l_, *x*_2_, ..., *x_n_* denote the possible interactive activities. Their corresponding probabilities also vary between 0 (no occurrence) and 1 (inevitable occurrence). These probabilities are known for the brain signal recordings, when we combine the adjacency relations in FCGB and the probability set in SE through Equations (1) and (2). It is reasonable to assume that the stronger the correlation between nodes, the higher its potential for functional integration and, consequently, its probability. According to SE, NE can be calculated as follows

(4) $$\mathrm{NE}=\sum_{i=1}^{n}p_{i}I_{i}=-\sum_{i=1}^{n}p_{i}\log_{2}p_{i},$$

where *I* is the information content of *X*. Formally, NE can be defined in terms of the expected (weighted average) amount of interactive information of *X*. To analyze the FCGB sequences, we rewrite Equation (4) as

(5) $${\mathrm{NE}}_{k}=-\sum_{i=1+m}^{L+m}p_{i}\log_{2}p_{i}$$

using the sliding window with the length of *L* (*L* = 10 × SL). Since the translation parameter has already been set to SL, the shift factor *m* = (*k* − 1) × SL indicates the amount of non-overlap (SL) during sliding. 

In epoch *k*, the probability distribution of SE {*p*_l_, *p*_2_, ..., *p_n_*} is replaced by the connection sequence distribution in ***F***. First, we create a correlation sequence {*γ*_l_, *γ*_2_, ..., *γ_n_*} from the upper triangular elements of ***F***, as the functional connectivity matrix is symmetric and the portion of ***F*** convey all the interactive information. To fulfill the requirement of $\sum_{i=1}^{n}p_{i}=1$, the correlation sequence is normalized. Then we have

(6) $${\mathrm{NE}}_{k}=-\sum_{i=1+m}^{L+m}\frac{\gamma_{i}}{\sum_{j=1}^{n}\gamma_{i}}\log_{2}\frac{\gamma_{i}}{\sum_{j=1}^{n}\gamma_{i}},\;\gamma_{i}\in \{\gamma_{1},\gamma_{2},\cdots ,\gamma_{n}\}$$

where *γ_i_* is an element in the correlation sequence $\sum_{i=1}^{n}\frac{\gamma_{i}}{\sum_{j=1}^{n}\gamma_{i}}=1$.

Due to the low SNR of ongoing EEG in single-trial traces [42], the wavelet time-frequency transform (see details in [43]) was utilized to resolve the FCGB sequence features {NE*_k_*} from two dimensions to carry out the event-related analysis of the changes in oscillatory activity in the final step of the NE algorithm. The wavelet-based transform is an effective tool for localizing the time-frequency components of a non-stationary signal because of its attractive properties, such as sharper time resolution in high-frequency components and multi-rate filtering (differentiating the signals that have various frequencies) [44]. In this paper, the “dmey” mother wavelet was selected for its efficiency in local representation. The sequence features {NE*_k_*} were decomposed between 0 Hz and 0.5 Hz with the scales of 1024. The frequency band of interest is 0–0.1 Hz.

### 2.2. Verification Experiment

In order to verify the effectiveness of the NE method, a driver fatigue experiment was conducted, in which various ongoing fatigue-related responses were defined as events. This part of the work aims at comparing the sequence analysis results of NE and the events happening in the experiment (true ground). Six healthy subjects (4 males, 2 females) with ages ranging from 26 to 39 years and a mean age of 29.8 were recruited. None of the subjects reported mental disorders, sleep problems, or neurological or developmental disorders. During the experiment, they were asked to drive along for one hour in a road environment simulating the suburban roads of Martinlaakso area in Vantaa, Finland, produced on the medium-fidelity driving simulator of the University of Jyväskylä. The driving simulator is equipped with a CKAS T2s2-DOF motion platform and three 40” LED displays for projecting the driving scene with 3420 × 900 resolution. Logitech G-25 steering wheel and pedals were used for controlling the simulated car.

In the prolonged driving task, two kinds of responses (with information also on timing) were recorded for validation purposes: self-reported subjective feelings and fatigue-related events. When the subjects felt fatigued, they self-reported this in real time by telling it to the researcher and the corresponding times were recorded by defining time-stamp to the data. Researchers also recorded observed fatigue-related events. When the subjects were observed to show the fatigue-related behaviors that they could not control such as, yawn, sigh, frequent blinking and doze, these were considered as objective fatigue-related events and the corresponding times were recorded by defining time-stamp to the data.

In the experiment, the EEG signals were collected by using the Wearable Sensing DSI-24 EEG measurement system. Nineteen dry electrodes (i.e., Fp1, Fp2, F7, F3, Fz, F4, F8, T3, C3, Cz, C4, T4, T5, P3, Pz, P4, T6, O1 and O2) mounted on a cap were attached to the scalps following the International 10–20 System to acquire data of brain activities. Pz was used as a reference electrode. The EEG data were digitized with the sampling frequency 300 Hz and sent to an external computer by wireless Bluetooth to be stored for offline processing. 

**Figure 2:**
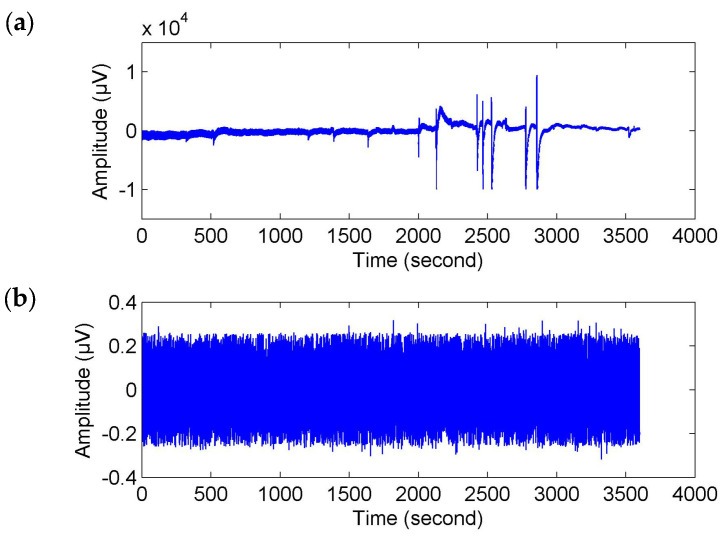
Preprocessing of original electroencephalpgram (EEG) signal. (**a**) Original EEG signal; (**b**) Corrected EEG signal.

Referring to Reference [32], the raw EEG data were filtered with a wavelet transform method between 18.75–37.50 Hz. Moreover, the wavelet-based threshold technique in Reference [32] was used to correct the filtered signals. Here, a stricter limiting condition was set up. If any of the wavelet coefficients *C* in the selected frequency band were greater than the threshold about mean and standard deviation mean(*C*) + 2 × std(*C*), it was set to zero to reduce the impact of artifacts. This conditional statement was executed twice. Then, the corrected EEG signals were obtained by reconstruction from the filtered coefficients (see Figure 2).

For statistical comparison of the global dynamic networks, five traditional network characteristics (i.e., clustering coefficients, characteristic path length, global efficiency and vulnerability) were calculated based on binarized connectivity matrices, which have a more easily defined null model for statistical comparison [45]. Here we construct binary brain networks using a thresholding approach. Without arbitrariness and priori assumption, a connectedness-based thresholding approach [46] was employed to achieve automatic binarization to discard weak and non-significant links. If the non-diagonal elements of the functional connectivity matrix ***F*** exceeded a threshold value *T*, they were set to 1 (otherwise 0). The threshold *T* was determined adaptively by choosing its maximal value such that the resulting network is connected (without an isolated node). Starting from *T* = 1, we gradually decreased the threshold (decrease step size = binarization precision 0.01) and we calculated, at each step, the second smallest eigenvalue *λ*_min_ of the corresponding Laplacian matrix ***L***. In a connected graph, the decreased step size had a relationship with the variances of the measured edges in ***F***. If the decreased step size was larger than the variances’ order of magnitude (i.e., insufficient binarization precision), the graph probably would not be a connected graph, no matter how threshold value *T* changed. In this paper, the decreased step size was selected by trial and error. We began with the order of magnitude of the edges’ standard deviation (0.01). If the binarization precision was insufficient, it would be decreased by an order of magnitude. The elements of the Laplacian matrix were obtained by the following equation

(7) $$L_{ij}=k_{i}\delta_{ij}-F_{ij},$$

where *k_i_* represents the degree of node *i*. *F_ij_* denotes elements of the functional connectivity matrix ***F***. *δ_ij_* is the function of Kronecker delta. 

(8) $$\delta_{ij}=\{\begin{array}{c} 0,i\neq j\\ 1,i=j \end{array}.$$

The threshold was determined, when *λ*_min_ became positive.

## 3. Results

**Figure 3:**
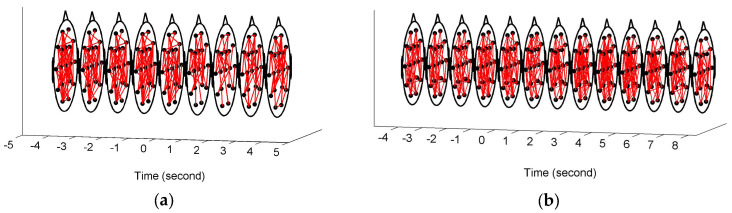
Event-related functional connectivity graphs of the brain (FCGB) sequences. (**a**) FCGB sequence corresponding to the response of a short sigh; (**b**) FCGB sequence corresponding to the response of a long sigh.

**Figure 4:**
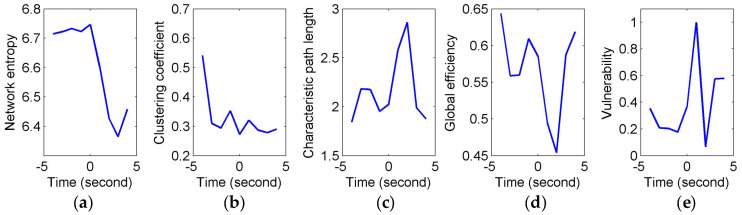
Comparison of event-related analysis results of a short sigh response. (**a**) Network entropy (NE) event-related analysis in the single trial; (**b**) Event-related analysis based on clustering coefficient in the single trial; (**c**) Event-related analysis based on characteristic path length in the single trial; (**d**) Event-related analysis based on global efficiency in the single trial; (**e**) Event-related analysis based on vulnerability in the single trial.

**Figure 5:**
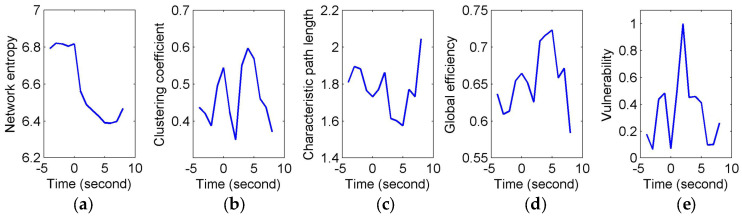
Comparison of event-related analysis results of a long sigh response. (**a**) NE event-related analysis in the single trial; (**b**) Event-related analysis based on clustering coefficient in the single trial; (**c**) Event-related analysis based on characteristic path length in the single trial; (**d**) Event-related analysis based on global efficiency in the single trial; (**e**) Event-related analysis based on vulnerability in the single trial.

Figure 3 shows the constructed FCGBs in two single trials involving two events: a short sigh in Figure 3a and a long sigh in Figure 3b. They were observed fatigue-related events. The FCGBs formed the graph sequences to perform the event-related analysis. In order to remove some artificial interactions and make the connectivity changes more obvious, the weighted FCGBs were binarized using the connectedness-based method. In Figure 3a,b, the origins of time axes represent event onsets. Two single trials from −4 s to 4 s and from −4 s to 8 s relative to the onsets of the short sigh and long sigh were extracted. The two FCGB sequences included the relative extrema in the temporal features (see Figure 4a and Figure 5a). Apparently, it is difficult to identify the features of the two events by visual inspection, even though the topological structure may contain useful interactive information.

To quantify and assess the topological properties of FCGB sequences, five network characteristics were calculated (see Figure 4 and Figure 5)—proposed NE, clustering coefficients, characteristic path length, global efficiency and vulnerability. The proposed NE versus time curves corresponding to the short and long sighs were plotted in Figure 4a and Figure 5a respectively. Other subfigures in Figure 4 (corresponding to the short sigh) and Figure 5 (corresponding to the long sigh) display traditional network characteristics, namely clustering coefficients (in Figure 4b and Figure 5b), characteristic path length (in Figure 4c and Figure 5c), global efficiency (in Figure 4d and Figure 5d) and vulnerability (in Figure 4e and Figure 5e). The clustering coefficients and characteristic path length constitute the small-world features. The lower clustering coefficients in Figure 4b and bigger characteristic path length in Figure 4c reflect the decrease of the small-world features after the event occurring. However, Figure 5b,c show the opposite trends, indicating the increase of the small-world features and more fluctuations indicating the low SNR and spurious interactions. The global efficiency has a similar situation with bigger fluctuations in the response of the long sigh. The vulnerability in Figure 4e and Figure 5e has a relatively consistent trend undergoing the same type of events but it is also affected by the disturbances and does not present stable differentiated information for the two events. Comparing with these traditional network characteristics, the proposed NE presents a coherent and stable trend associated with the event occurrence. At the same time, the distinct downward NE changes in Figure 4a and Figure 5a reflect the characteristics of the two events (short and long). It reveals that the interactive information extracted by NE can be time-locked and consistent with the actual objective responses to some extent. 

**Figure 6:**
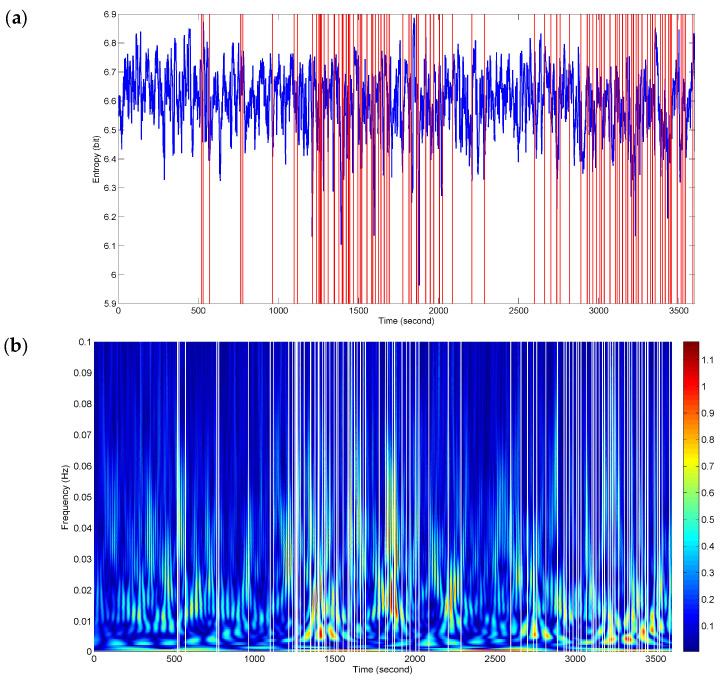
Sequence analysis results of NE in the whole duration of driving task. (**a**) NE versus time curve; (**b**) NE time-frequency features.

In order to detect as many of the fatigue-related responses as possible during driving, NE was applied to assessing the complexity of interactive activities from the complete 1 h FCGB sequences. Figure 6 presents sequence analysis results of NE for the whole driving process. The red vertical lines represent the timing of the recorded subjectively and objectively reported markers of fatigue (described in Section 2.2). The two events in Figure 4 and Figure 5 are included in them. The NE versus time curve in Figure 6a demonstrates the time-locked features just in a few events. Here, the time-locked feature represents a time component repeatedly emerging with the same onset time. Nevertheless, the density and downward trends of NE seem to match the two stages (ranging from 1096 s to 2096 s and from 2602 s to 3600 s) of dense event distributions. It provides a bright prospect for further time-frequency analysis. 

Figure 6b shows a dramatic improvement for the time-locked features obtained by time-frequency analysis of NE. The white vertical lines represent the recorded responses. The high power changes (red-biased spectra) have a certain relevance to all the events of the whole process in terms of a relatively high degree of correspondence. At the stages of dense event distributions, the time-frequency features have a wide range of high-power activities in the low frequency band below 0.01 Hz, which reveals the physiological fatigued states. Meanwhile, the high frequency components also retain the localization information. 

**Figure 7:**
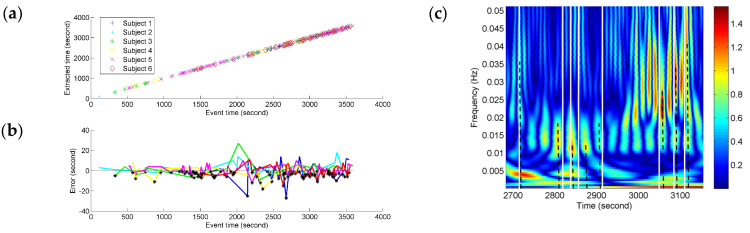
Comparison between the extracted time based on NE and the recorded event time in the experiment. (**a**) Correlation between the extracted event time and the recorded event time; (**b**) Errors between the extracted event time and the recorded event time; (**c**) Event time extraction based on the NE time-frequency features.

Depending on the precision of the time-frequency localization based on NE, the localized time of the FCGB sequence features from all the subjects was extracted and compared with the recorded event time in the experiment (see Figure 7). In Figure 7c, the partial enlargement of Figure 6b demonstrates the extraction process of the localized time. The nearest high power activities from the recorded events were considered as event-related activities. The time was determined by the centerlines of the high power activities. The centerlines are represented as the dotted lines in Figure 7c.

We tested for the correlation between the extracted time based on NE and the recorded event time by using Pearson correlation coefficient. A significant correlation was found (*r* = 0.99997, *p* < 0.001) for different fatigue responses for all the subjects. As shown in Figure 7a, the fatigue-related event times and the estimated times for these have a high degree of match and the time points with the horizontal and vertical coordinates (recorded event time, NE-based extracted time) can be fitted by a line. The time points are denser at the end stage of driving indicating more fatigue-related events. To quantify the variances of NE measurement compared to the fatigue-related event times, the NE-based outputs’ time errors from the event recordings were calculated and are shown in Figure 7b. Since the extracted time based on NE was used as minuend, the black points below the *x*-axis (zero error) illustrate the situation of NE-based extracted time being before the recorded fatigue-related event time. The color of an individual error line refers to the colors in Figure 7a. All the time errors are distributed within the range from −30 s to 30 s and the biggest errors for different subjects seem to be distributed around 2000 s which is near the mean (17,876 s) of the subjective fatigue-related event times. About ninety percent (90.1%) of the time errors are distributed within the range from −10 s to 10 s (roughly equal to the duration of a long sigh response).

## 4. Discussion

Changes in functional connectivity alongside changes in mental states can provide richer information about human cognition than simpler univariate approaches [13]. However, the information is usually implicit in a complex process because of interference from various external factors. Especially for the single-trial analysis of spontaneous EEG, the useful components cannot be enhanced through the average of repeated events. During the dynamic network analysis, false positives probably occur for the FCGB estimation at the level of the EEG sensors.

Therefore, ‘double insurance’ measures were taken in this study. For one thing, NE was proposed to assess uncertainty in the connectivity matrices of FCGB sequences. Signal mixing did not vary over time [25,47,48,49,50]. Referring to law of conservation of matter and energy, if the physiological state or source activity did not change considering external factors, the total amount of information that could be detected was unchanged, no matter how the signals were leaked and mixed. The amount of information (i.e., uncertainty of information sources) was measured by NE. That was the principle that NE alleviated the spurious interaction problem in the FCGB sequence analysis. For another, NE oscillation activities were extracted to optimize the dynamic analysis in time-frequency domain. Signal mixing did not vary across frequency bands [25,47,48,49,50]. The NE features were resolved in the time-frequency domain to further reduce the multivariate mixing effects and fluctuations in single dimension information. The event-related analysis of spontaneous EEG would be promoted as a result of the time extraction of NE oscillation changes extending over frequency bands. These NE oscillations had hardly any correlation with the false positives of the functional connectivity measures.

In this paper, FCGBs were constructed by using the edge measurement method in the time domain (i.e., cross-correlation function). As shown in Figure 3, FCGB sequences were formed with the employment of a sliding window to achieve the single-trial analysis. In Figure 4 and Figure 5, the topological properties of FCGB sequences containing the dynamic information of interactions were quantified by the proposed NE method and four traditional methods. The traditional analysis methods based on graph theory generally extract the network characteristics, which are related to the variables of the number of links in the nodes or the weights of edges [9]. The small-world features, global efficiency and vulnerability adopt the principle of functional segregation and integration to reflect the brain’s ability to efficiently integrate information, the efficiency of information exchange and resilience [13,15]. Figure 4 and Figure 5 depict a specific situation for two transient events representing moments of fatigue, namely short and long sighs, respectively. These network characteristics obtained using traditional methods seemed not to be sensitive to the changes in fatigue during driving in this study so that the critical information of the two events was hidden. It was relatively difficult to identify the “short” and “long” sighs in Figure 4b–e and Figure 5b–e with redundant information. Figure 4b,c and Figure 5b,c even present opposite trends after the event occurrence. One possible explanation is that external factors have a serious impact on the outcomes and their own ability is limited by the connection-based or weight-based assessment. 

Compared to the traditional methods, the proposed NE method showed robustness in the FCGB sequence analysis owing to the introduction of the entropy concept from information theory. It demonstrated time-locked features in Figure 4a and Figure 5a for the two events and retained the clearer “short” and “long” changes. It provides the possibility of separating short sighs from long sighs in the individual epochs. As a measure of average uncertainty of event set, the entropy value will be higher when the system is more complex and disordered. The NE valleys after the event occurrence reflected the fact that the information amount of interactive activities reduced and processing capabilities of brain networks decreased when mental fatigue made the subject show visible symptoms of fatigue. Note that sighs have a function of relief from a psychological perspective and they reset breathing variability and oxygenation [51]. Nonetheless, we focus on the weakening effect of fatigue in the prolonged driving task. The subjects may also not call the recovered resource. As reported by the subjects, they were reluctant to spend energy on this task any more, when the behavioral events occurred. Additionally, these behavioral events are associated with or are indicative of certain emotional states and, with the exception of relief, mostly negative ones [52]. This negative impact combined with fatigue’s negative impact may cause the NE decline.

Of course, for fatigue related behavioral events (e.g., yawns and sighs), where there were movements of the subjects, they probably caused movement artifacts (or muscle artifacts) in the EEG signals (see Figure 2a). To make sure that the time-locked features were not caused by muscle artifacts, we used the wavelet-based threshold technique with a relatively strict limiting condition to correct the EEG signals. As shown in Figure 2b, the corrected EEG signal got closer to a stationary signal but it was much weaker than the original signal and the usual EEG signal. We may also lose a large part of the cerebral activity. Hence, the event-related analysis (Figure 4, Figure 5, Figure 6 and Figure 7) seems to be more important. In addition, no matter what form of artifacts (e.g., ocular, cardiac and muscular artifacts), the SNR or power is much higher than that in EEG. In other words, their appearance will generate an enhanced effect in the individual epochs. Conversely, fatigue has a weakening (or negative) effect on physiological function. Thus, the temporal features with a downward trend in Figure 4a, Figure 5a and Figure 6a were considered to be a reflection of fatigue patterns.

Nevertheless, throughout the whole driving process, the performance of NE degraded in the plentiful single-trial traces. Only a few changes matched the events in Figure 6a. The NE features just in time domain did not yet reach the practical requirements. It is not possible to set a threshold or extract systematic patterns. One of the reasons may be that several factors affect functional brain activities throughout the complex process and the factors may not only come from the external environment but also from internal activities. Driving involves various functions such as movement, perception and recognition, visual and auditory processing, reasoning and decision-making [32]. The level of fatigue was determined by using subjectively or objectively recorded events reflecting feelings of fatigue or fatigue related behaviors. There are probably implied overlapping events in the recordings. Due to the nature of cognitive processes, the feature pattern at each event may not be as straightforward as complexity increasing or decreasing. In addition, the spurious interaction issue may not be completely resolved by NE in a single dimension.

On the basis of this, the time-frequency transform of NE was performed to realize the event-related analysis in the time and frequency dimensions, with a smooth enough temporal precision for observing cognitively relevant changes. As shown in Figure 6b, the time-frequency method resolved certain critical temporal information in the high frequency band and also output a wide range of high-power activities in the low frequency band. The timing of these activities overlaps with the fatigue stage in which the recorded behavioral events occurred intensively. The improvement of the time-locked performance supports the hypothesis that the dynamic interactive activities of FCGBs contain rich information for event-related analysis in single-trial traces but the information is implicit. The NE time-frequency features are helpful for distinguishing physiological states and detecting critical fatigue. It is important to note the possibility that those high-power activities can still be contaminated by the residual artifacts.

Comparison between the extracted time based on NE and the recorded event time in the experiment further demonstrated the potential of the quantitative NE-based analysis (see Figure 7). When extracting the time-course of high power activities based on the NE time-frequency features, there was always a high-power activity near the recorded fatigue-related event (see Figure 7c). Hence, through Pearson correlation coefficient calculation, *p* < 0.001 between all the recorded event time and the extracted time revealed a statistically significant correlation. That means the high-power activities of NE have a high degree of correspondence with the event occurrence. The time points of fatigue related events are denser at the end stage of driving indicating increasing fatigue with task duration. At the end stage of driving with denser event distribution, more high-power activities burst. It seems that the NE oscillation features from the FCGB sequence are prominent fatigue indicators. Figure 7b showed the variances of NE measurement compared to the fatigue-related event times. All the time errors in Figure 7b were distributed within the range [−30 s, 30 s] and 90.1% of the time errors were distributed within the range [−10 s, 10 s], which also showed the effectiveness of the proposed method. The largest errors of all the subjects were distributed around 2000 s in Figure 7b. Therefore, these large errors may not be caused by the method drawback but point to common state changes. Besides, 110 (black points) out of 232 the NE high-power changes preceded the recorded fatigue-related events. Since the high-power changes were obtained by the time-frequency transformation of NE, they had a relationship with the high-density NE decline (in Figure 6a). As mentioned above, high-power activities may also be contaminated by residual artifacts. These early high-power changes, determined by the black points in Figure 7b, provide useful information for further reducing the influence of residual artifacts to research the physiological state changes and developing the early warning system of driver fatigue in practical applications.

For dynamic network analysis, the most common approach has been the sliding-window technique and previous studies have pointed out its shortcomings, including the arbitrary choice of window length and the fact that all observations within the window are weighted equally [41,53,54]. As mentioned above, the edge measurement of cross-correlation also suffers from signal leakage [26]. Thus, the ability to assess dynamic changes in network properties is critical for a better understanding of brain activities. To assess uncertainty and avoid spurious fluctuations in the dynamic process, Kudela et al. utilized a multivariate linear process bootstrap method for obtaining non-parametric estimation of the confidence bands for the dynamically changing correlation coefficient [41]. It provided a feasible route for sliding-window correlation estimates to address one of the main issues. Furthermore, we introduced SE to directly calculate the uncertainty of functional connectivity in the FCGB sequences determined by the sliding window to conduct the dynamic network analysis. Note that what would be an optimal window length for a specific EEG signal and should we use windows with equal or unequal lengths are still unanswered questions. The sliding-window parameters (10 × SL window length and SL shift) just meet the requirements of the second-scale event analysis in the prolonged driving task.

Overall, the single-trial connectivity analysis itself has a certain degree of difficulty, limited to the low SNR of EEG. It is a challenge to realize event-related analysis of spontaneous EEG in a complex process without stimuli and average but with signal mixing/leakage. In this paper, the proposed NE method for the sequence analysis of FCGB offers a possible solution to this problem. Particularly, the high-power activities of NE in the time-frequency domain had a high correlation with all the fatigue-related events in the verification experiment. It demonstrates potential value for the study of cognition and for detecting various driver fatigue-related events in practical applications.
